# A Two-Dimensional Pooling Strategy for Rare Variant Detection on Next-Generation Sequencing Platforms

**DOI:** 10.1371/journal.pone.0093455

**Published:** 2014-04-11

**Authors:** Philip C. Zuzarte, Robert E. Denroche, Gordon Fehringer, Hagit Katzov-Eckert, Rayjean J. Hung, John D. McPherson

**Affiliations:** 1 Genome Technologies, Ontario Institute for Cancer Research, Toronto, Ontario, Canada; 2 Lunenfeld-Tanenbaum Research Institute of Mount Sinai Hospital, Toronto, Ontario, Canada; 3 Department of Medical Biophysics, University of Toronto, Toronto, Ontario, Canada; 4 Division of Epidemiology, Dalla Lana School of Public Health, University of Toronto, Toronto, Ontario, Canada; Deutsches Krebsforschungszentrum, Germany

## Abstract

We describe a method for pooling and sequencing DNA from a large number of individual samples while preserving information regarding sample identity. DNA from 576 individuals was arranged into four 12 row by 12 column matrices and then pooled by row and by column resulting in 96 total pools with 12 individuals in each pool. Pooling of DNA was carried out in a two-dimensional fashion, such that DNA from each individual is present in exactly one row pool and exactly one column pool. By considering the variants observed in the rows and columns of a matrix we are able to trace rare variants back to the specific individuals that carry them. The pooled DNA samples were enriched over a 250 kb region previously identified by GWAS to significantly predispose individuals to lung cancer. All 96 pools (12 row and 12 column pools from 4 matrices) were barcoded and sequenced on an Illumina HiSeq 2000 instrument with an average depth of coverage greater than 4,000×. Verification based on Ion PGM sequencing confirmed the presence of 91.4% of confidently classified SNVs assayed. In this way, each individual sample is sequenced in multiple pools providing more accurate variant calling than a single pool or a multiplexed approach. This provides a powerful method for rare variant detection in regions of interest at a reduced cost to the researcher.

## Introduction

Genome wide association studies (GWAS) provide a wealth of information about the genetic basis of disease. As regions of the genome that are involved in pathogenesis are identified there is a need for improved fine mapping of genetic variants associated with disease over a large number of individuals. Re-sequencing of GWAS peaks offers the potential to identify rare variants within regions of interest however the complexity and cost of sequencing large number of samples remains prohibitive.

Sample pooling is a frequently applied method for sequencing a large number of samples in order to detect variants. Targeted enrichment of specific regions of interest prior to pooling can increase the number of samples processed using current sequencing technologies. Bioinformatics tools such as VarScan and CRISP exist for single nucleotide variant (SNV) calling from pooled samples but are not capable of identifying the specific samples in the pool that contributed the variant [1] [2]. Sample barcoding may be applied in order to allow sample identification but this approach forgoes the cost benefit of a pooled library preparation. Thus, improved methods are required to enable degrees of sample deconvolution for DNA that is pooled prior to library preparation for sequencing.

Multi-dimensional pooling of samples offers a powerful solution to this problem. By pooling samples along different dimensions and then considering the commonalities between the variants called in each pool the cost savings benefits of pooled library preparation are leveraged while the ability to identify the specific individuals that possess a variant is retained. Multi-dimensional pooling strategies have previously been used to increase throughput of large-scale genomics projects while reducing the cost of handling large amounts of samples. Notable examples of this strategy include approaches for identifying pooled Bacterial and Yeast Artificial Chromosomes in cloned arrays by probe hybridization or PCR [3] [4]. Keypoint technology uses targeted re-sequencing of PCR products pooled in 2 or more dimensions and ‘DNA Sudoku’ has been described for sequencing very large numbers of pooled bacterial clones containing short sequences encoding shRNA [5] [6]. TILLING (Targeting Induced Local Lesions IN Genomes) has also been applied in mutagenesis and reverse genetics studies where the target gene is known [7]. These methods are effective strategies for sequencing multi-dimensionally pooled samples, however none describe an application to target-enriched, next-generation sequencing. Furthermore, these approaches prioritize preserving the ability to determine the contributing source of every observed feature – a requirement which is more tractable in rare variant detection.

**Figure 1:**
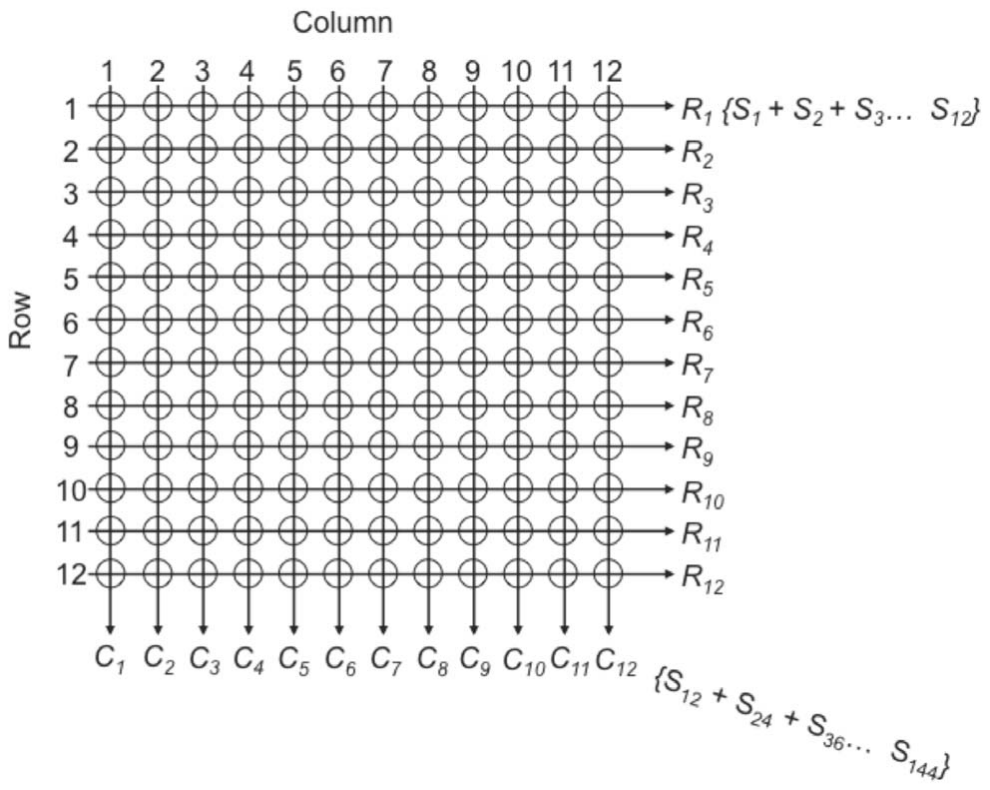
Row by column arrangement for pooling of DNA samples. 576 DNA samples (S*_n_*) were arranged in six 96 well plates. These samples were pooled in 4 matrices of 12 rows by 12 columns as illustrated. For each pool, 12 DNA samples were either pooled across the grid (row) or down the grid (column). Each pool of 12 DNA samples was then target enriched, barcoded and processed for Illumina sequencing.

We describe a two-dimensional pooling method which we used to identify rare variants in 576 individuals over a 250 Kb region previously identified by several genome wide association studies to significantly predispose individuals to lung cancer [8]–[12]. DNA from the 576 individuals was arranged into 4 matrices, each containing 12 columns and 12 rows (Figure 1). The pooled samples were subsequently enriched for DNA from the region of interest. Pooling of DNA was carried out in two dimensions such that when a variant is reported in exactly one row pool and one or more column pools, or exactly one column pool and one or more row pools, we are able to identify the individual carrying that variant. Additionally, because each individual sample is sequenced twice, two independent measurements are made of each variant which increases the accuracy of variant calling. With this approach, a large number of samples may be sequenced to great depth over an enriched target area. This provides a powerful method for rare variant detection in regions of interest at a reduced cost to the researcher and with high verification rates.

## Results

### Pooling and sequencing

Pooled DNA was captured using custom probes and Agilent SureSelect technology [13] targeting a 250 kb region of chromosome 5 (5p15.33). Captured DNA was prepared for Illumina sequencing (as described in the Methods section) such that a pool of barcoded rows and a pool of barcoded columns was created from each matrix (12×12) over a total of 4 matrices.

The 8 pools were sequenced on a HiSeq Illumina instrument resulting in very deep coverage of our 250 kb target (Table 1). The raw sequencing yield for each pool of row and column libraries exceeded 24 gigabases with an average of 72% of the reads mapping to the human genome. Among reads mapping to the human genome, an average of 74% mapped to our targeted region and each row and column was covered to an average depth greater than 4,000× (Table 1). Coverage of sequencing across the region tended to be evenly distributed; however, a few region-specific tandem repeats were captured resulting in peaks of ambiguously mapped sequence (as can be seen in Figure S1 in Supplemental File S1). Regardless, upwards of 75% of the target region was covered at a depth of at least 200× (our minimum depth threshold for calling variants in these pools) representing at least 16 reads per individual if we assume even pooling.

**Table 1:** Sequencing yield and efficiency of enrichment.

Pool	Raw Yield (Gb)	% Mapped	% On Target (of mapped)	Total Coverage in Region	Average Coverage per Pool	Average Coverage per Sample
Matrix 1 Columns	25.41	64.66%	78.98%	51,840	4,320	360
Matrix 1 Rows	26.53	66.29%	75.97%	53,424	4,452	371
Matrix 2 Columns	41.37	75.10%	74.62%	92,736	7,728	644
Matrix 2 Rows	28.74	73.36%	71.17%	60,048	5,004	417
Matrix 3 Columns	70.86	79.64%	72.13%	162,720	13,560	1,130
Matrix 3 Rows	29.87	76.59%	78.81%	72,144	6,012	501
Matrix 4 Columns	25.36	68.12%	68.12%	52,416	4,368	364
Matrix 4 Rows	24.33	73.20%	73.20%	54,144	4,512	376

The raw yield, percentage of reads mapped, percentage of reads on target, the total coverage of the region and the average coverage per pool and per individual is shown for each 12 row and 12 column pool from each of the four matrices sequenced.

### Calling and classifying variants

**Figure 2:**
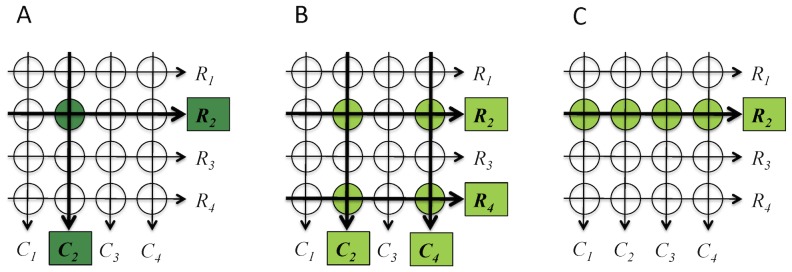
Definition of variant classes. Variant calls are classified based on their relationship to the pooled individuals. The three possible classes are *Pinnable*, *Multiple* and *Singleton*. (A) *Pinnable* variants were those where the carrying individuals may be identified because there is exactly one row or exactly one column containing a variant and at least one intersecting row or column pool. (B) *Multiple* variants were those where the variant is observed in more than one row and more than one column and it is not possible to determine precisely which individuals possess the variant. (C) *Singletons* were calls that are only observed in either a row or a column but not both.

Reads were demultiplexed, aligned, filtered for quality, and analyzed as detailed in the Methods section. Variant calls were determined individually in each of the row and column pools using base counts in samtools pileup files [14]. Then variants were considered across all pools in order to classify the call based on whether it could be traced back to the specific individuals who carried the variant. Calls with sufficient coverage were classified as one of the following: *pinnable*, *multiple* or *singleton* (Figure 2). Variants that fell below our cutoff of 200× coverage in 3 or more pools were classified as *missing coverage* variants.

*Pinnable* variants were those that could be attributed to a specific individual because the variant was called in only one row and one or more columns, or only one column and one or more row pools of a matrix (Figure 2A). We observed between 312 and 425 *pinnable* class single nucleotide variants in each matrix resulting in a total of 1260 unique *pinnable* SNVs across all four matrices (Table 2). Variants that were called in more than one row and more than one column pool were classified as *multiple* (Figure 2B). We identified an average of approximately 625 *multiple* class SNVs in each matrix, of which very few were private to one matrix; in total over the four matrices we found only 697 unique *multiple* SNVs. A third class of variant was termed a *singleton*. *Singletons* were observed in either a row or column pool but not in both (Figure 2C), which was unexpected given the design of the experiment, and suggest either a false positive in the observed pool or a false negative in at least one intersecting pool. Nevertheless, we called between 179 and 341 SNVs of this class per matrix, and found 864 unique *singleton* SNVs in total.

**Table 2:** Summary of SNV class by matrix.

Matrix	Pinnable Variants (% dbSNP)	Multiple Variants (% dbSNP)	Singleton Variants (% dbSNP)
1	371 (22.64%)	591 (82.40%)	341 (5.28%)
2	425 (28.53%)	613 (81.24%)	179 (11.17%)
3	408 (23.04%)	680 (78.97%)	245 (6.94%)
4	312 (17.95%)	621 (81.80%)	231 (3.46%)
Total unique	1260 (20.87%)	697 (74.18%)	864 (2.89%)

For each 12 row by 12 column matrix of libraries sequenced, the number of *Pinnable*, *Multiple* and *Singleton* single nucleotide variants is given. Also indicated is the percentage of each variant class that is catalogued in dbSNP. The number of total variants for each of the classes represents the total unique number of variants from all four matrices.

**Figure 3:**
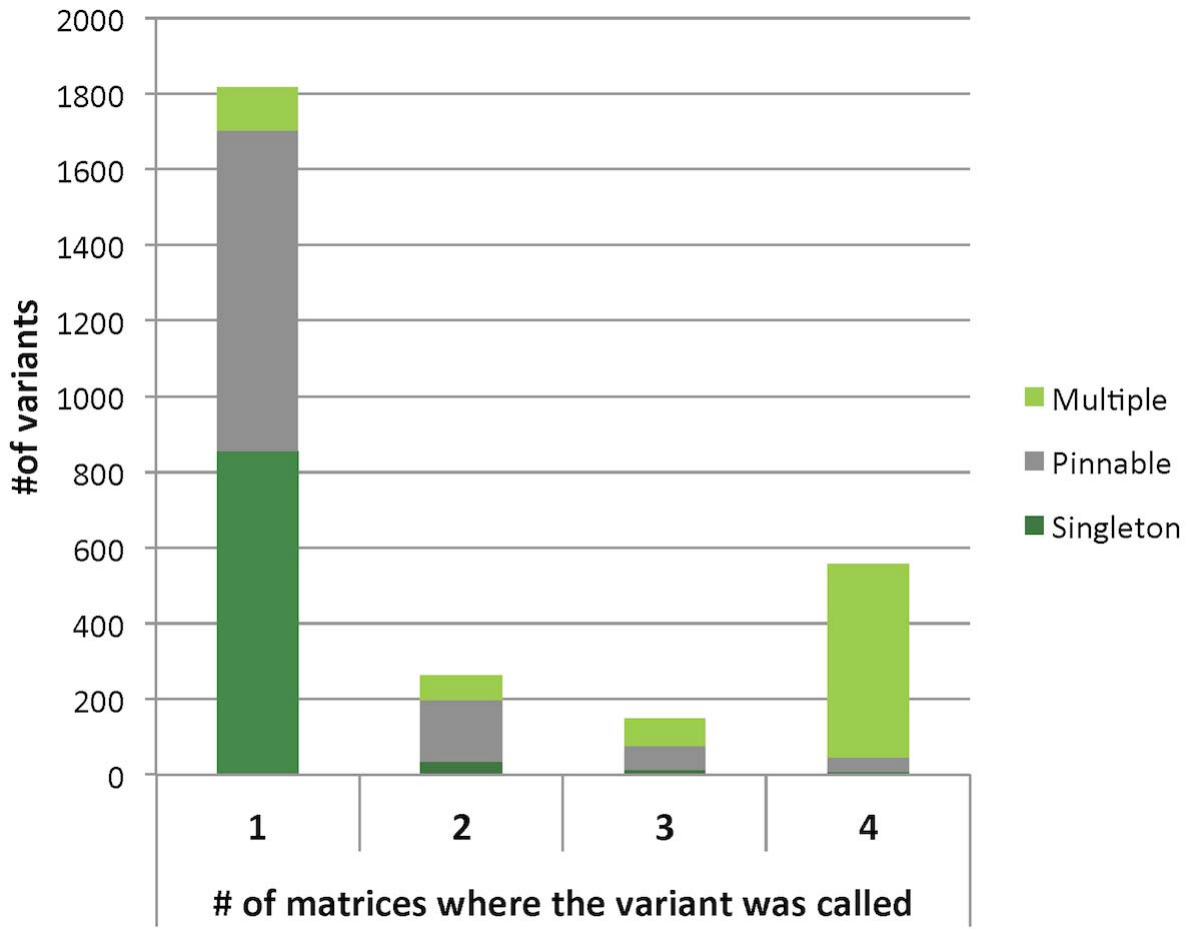
Bar graph of variant classes by frequency of observation. A breakdown of the classifications of variants that were observed in exactly 1, 2, 3 or 4 of the 4 matrices. Variants that were rare within a matrix (and thus labeled *Pinnable* or *Singleton*) were predominantly seen in only 1 of the 4 matrices. Similarly, variants that were common within a matrix (*Multiples*) were also common between the 4 matrices.

DNA was arranged into matrices without prior knowledge of the specific variants that the individuals possessed, therefore we expect that rare variants in a matrix (*e.g.* a *pinnable* that only occurs once in a 144 position matrix) should also be rare in the study, and rare in the population. Likewise, a variant that occurs commonly in a matrix (*e.g.* a *multiple* that is observed in several rows and columns) should also be common in the study as well as in the population. Figure 3 shows the number of SNVs that were called in exactly 1, 2, 3 or 4 matrices broken down by classification. The *pinnable* and *singleton* variants that were rare within one matrix were also predominantly only called in one matrix - over 90% of the variants that were unique to one matrix fall into these two classes. Furthermore, if the presence of a variant in dbSNP 132 is indicative of how common a variant is in the population, we see that only 20.87% of the *pinnable* and 2.89% of the *singleton* SNVs were present in dbSNP (Table 2). Thus, variants that appear to be rare in one matrix were also rare in the study and in the population. The converse also holds; Figure 3 shows that most *multiple* class SNVs were called in all 4 matrices and that over 90% of SNVs that were seen in every matrix were *multiples*. Additionally, Table 2 reports that 74.18% of the *multiple* SNVs could be found in dbSNP. As expected, the *multiple* class variants that were common within a matrix were also common across our study and in the population.

### Verification results

An amplicon sequencing approach was taken to verify variants predicted using our pooling approach. We selected candidate SNVs to verify using two methods; by random selection from a list of all variants with a given classification and also based on their predicted consequences because we were most interested in non-synonymous changes and SNVs that may affect splice sites. Consequences were predicted by ANNOVAR [15] (see Table S1 in Supplemental File S1). Primers were designed to amplify short stretches of DNA containing the selected variants. Amplicons were indexed, pooled and then sequenced on an Ion Torrent Personal Genome Machine.

As detailed in Table 3, we assayed 58 *pinnable* SNVs (18 of which were selected due to their predicted consequence) and found that 53 calls were true positives (91.4%) in the specific individual indicated by the intersection of the row and column pools where the SNV was observed. SNVs that were observed as *multiple* were verified in two different ways; (1) Verification of the variant within a pool of DNA (an entire row or column) and (2) Verification of every DNA sample at the intersection of called pools (individual DNA samples predicted to contain the variant). Of the 8 SNVs chosen randomly for verification in their DNA pool, 7 were confirmed. These variants (7/8) were confirmed in pools of DNA containing all 12 DNA samples from a row or column where the variant was called. An additional 17 *multiple* SNVs (12 selected at random and 5 hand chosen based on consequence) were tested where each individual DNA sample was assayed at the intersection of all row and column pools predicted to contain the variant. Of these 17 SNVs, 13 were confirmed. The combined verification rate for the two methods of *multiple* verification was 80%.

**Table 3:** SNV verification rates by class.

Class	Variants in Class	Randomly Selected Verification Rate	Consequence Selected Verification Rate	Total Verification Rate
Pinnable	1,260	36/40 (90.0%)	17/18 (94.4%)	53/58 (91.4%)
Multiple	697	16/20 (80.0%)	4/5 (80.0%)	20/25 (80.0%)
Singelton	864	0/7 (0.0%)	1/5 (20.0%)	1/12 (8.3%)

Candidate variants of each class were selected for verification using an orthogonal sequencing chemistry. DNA samples predicted to carry specific variants were PCR amplified and sequenced on an Ion Torrent PGM instrument.

We identified 12 *singleton* SNV candidates for verification (including 5 due to their predicted consequence) and performed PCR on each individual from the row or column where the variant was detected. Only 1 of the 12 *singletons* assayed was confirmed to be a true positive in the row (and thus a false negative in the column) and the rest were shown to be false positives. The likelihood that *singletons* were primarily false positives was supported by the low number of reported calls that were found in dbSNP (Table 2) and by the observation that the 1 verified *singleton* was the only variant of the 12 tested to be found in that database.

Detection of indel variants was also possible with the two-dimensional pooling approach we have described. We identified 70 *pinnable*, 78 *multiple* and 150 *singleton* indel variants in our dataset (dbSNP rates and consequence calls are available in Tables S2 and S3 in Supplemental File S1). Initial verification of a small subset of 10 *pinnable* indels selected at random found that 9 were indeed true, which suggests a true positive rate similar to what was observed for *pinnable* SNVs.

## Discussion

Two-dimensional pooling has proven to be an effective strategy for the detection of rare variants in targeted next generation sequencing. Pooling strategies have a clear cost benefit over preparing each sample individually. However, with a typical one-dimensional pooling it is impossible to determine which sample contributes to each variant without extensive barcoding of samples requiring individual sequencing library preparation. Pooling samples in two dimensions enables the rare variants in a matrix to be traced back to specific individuals pooled in a matrix.

We identified a number of SNV candidates in our experiment, including 1,260 *pinnable* classed variants that could be assigned to an individual DNA sample in the 576 analyzed. An additional 697 *multiple* classed variants were observed in multiple rows and columns. Verification results have shown that a very high number of the *pinnables* were truly present in the individual specified by the call. When verifying *singleton* variant candidates, we found that in a majority of the cases the initial *singleton* call was a false positive, and conclude that calls from this class can be filtered from the results.

The high number of *singleton* calls that were verified to be false is indicative of an important benefit two-dimensional pooling has over one-dimensional approaches; DNA from each sample is sequenced twice in two different libraries and variants must be detected in both to make a positive call. In one-dimensional approaches, *singleton* calls would appear identical to other positive calls. In two-dimensional pooling, however, these calls were clearly *singletons* and can be filtered accordingly, improving the true positive variant detection rate of the experiment.

One drawback of the two-dimensional approach is that once a variant is detected in more than one row and more than one column it becomes impossible to determine precisely which individuals possess the variant. However, it is possible to narrow down the list of individuals; at least one individual from each observed row or column, and at most every individual at the intersections of the observed rows and columns may carry the variants. By increasing the number of pooling dimensions, approaches such as DNA Sudoku can allow common variants to be traced back to the contributing individuals at the cost of additional library preparation and sequencing.

Two-dimensional pooling and sequencing allows the identification of rare variants in a targeted region with more accuracy than traditional one-dimensional sequencing (because each variant in each individual is sequenced twice). Pooled sequencing in more than one dimension can be carried out at a fraction of the cost of capturing, indexing and sequencing each individual separately, while retaining the ability to identify individuals possessing rare variants. For applications where the number of samples are high and the variants of interest are likely to be rare we have shown two-dimensional pooling to be an effective approach.

## Materials and Methods

This research was performed with the approval of the Mount Sinai Hospital (Toronto, Canada) Research Ethics Board (#07-0167-E). Signed consent was obtained from adult participants. No children were included in this study.

### DNA Pooling, Capture and Sequencing

DNA aliquots from 576 individuals were arranged into 4 different 12×12 matrices and then pooled by row and by column as shown in Figure 1. Each pooled row and column contained DNA from 12 different individuals. Each of these pools were processed using a custom Agilent SureSelect Indexing kit designed to capture and generate indexed Illumina-compatible libraries enriched for a 250 kb region of 5p15.33. Genomic capture and library construction using the Agilent SureSelect system was performed as recommended by the manufacturer with a hybridization capture time of 72 hours.

Captured and indexed libraries were multiplexed into 8 pools and sequenced on 8 lanes of an Illumina HiSeq instrument. Due to variations in pooling, under-performing libraries were re-sequenced on an additional lane. Sequencing was carried out as described by the manufacturer (Illumina) generating 2×101 bp indexed pair-end reads.

### Initial data processing

We called bases and generated demultiplexed fastq files using Illumina's CASAVA pipeline. Reads were aligned using Novoalign V2.07.06 (www.novocraft.com) and any reads with a mapping quality less than 30 were deemed to be not uniquely aligned and were discarded. Next, we locally realigned reads using version 1.0.5083 of the Genome Analysis Tool Kit [16]. Any read with more than 2 mismatches was discarded. We did not remove potential PCR duplicates because each pool contained reads from multiple individuals and it was important to maintain the relative allele frequencies present in the data. Detection of PCR bias was handled later by a metric built into the variant calling step.

### Variant calling

Samtools and downstream filtering were used to call single nucleotide variants (SNVs) in each individual pool based only on high quality (q> = 30) bases at positions that did not fall into UCSC's repeat mask or self chaining tracks. Any position where there were fewer than 200 reads available or when there was potentially a start point bias (a score of less than 1.25 using our metric, which is described next) was marked as having insufficient coverage and was excluded in the calls for the pool. The depth and start point bias cutoffs were selected by using the percentage of variants called that were reported in dbSNP as an indication of our true positive rate.

Our start point bias metric was based on the number of unique start points contributing to a call, as well as the distribution of the coverage granted by each start point. If the majority of the reads originate from one start point, the position would receive a low score on the metric. If *d_i_* is the number of reads that start at the *i*-th start point and *n* is the total number of start points, then the metric is calculated as follows:
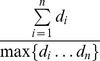



We consider the percentage of reads that support a non-reference base and we applied the following criteria in order to make one of 4 possible calls at every position with sufficient coverage. It is important for our method to call both the presence and absence of variants because knowing that a variant is not in a pool is crucial for determining if the variant can be traced back to the individual that carried it. First, a *confident* variant is called at positions where greater than 1% of the reads support the variant and we have sufficient coverage. A *potential* variant was called when more than 0.5% of reads supported the variant. We make a *potential* call for the absence of the variant if fewer than 0.5% of reads support the variant. Finally, we consider a position a *confident* no-variant call when fewer than 0.1% of reads support the variant.

### Variant classification

Once variants have been called in the rows and columns of a matrix, only variants that had at least one *confident* call (as defined above) were included; if a variant only had *potential* calls supporting it then it was discarded at this stage. By considering the number of row or column pools where a variant was called (whether *confidently* or *potential*) as well as the number of pools the reference was called in, each variant could be classified as follows:

1. A variant was classified as *pinnable* when it was observed in exactly one row and one or more columns, or in exactly one column and one or more rows. *Pinnable* calls could be traced back to the individual in the matrix who carry the variant by considering the intersection of the row and column where the variant was observed.
2. Variants were classified as *multiples* when they were observed in more than one row and more than one column. From a *multiple* call, we know that at least some of the individuals at the intersections of the rows and columns carried the observed variant, but it is impossible to determine exactly which individuals have contributed the variant to the pools.
3. If we observed a variant in a row but not in a column, or in a column but not in a row, then we classified the variant as a *singleton*.
4. In order to differentiate between the above classifications, we need to know exactly how many row and column pools a variant has been observed in. If more than 3 pools had insufficient coverage, we did not have enough evidence to accurately classify the variant. We report these variants under the *missing coverage* class. In Text S1 in Supplemental File S1, we provide a pseudocode listing of the processing steps and classification algorithms used in this study. Perl scripts will be provided upon request, but may require modification to work in other computing environments and with other datasets.

### Variant Verification

SNV candidates were selected for verification in two ways – at random and by examining consequence calls generated by ANNOVAR (see Table S1 in Supplemental File S1). Primers targeting each variant were designed using a Primer3 based script. The primers were barcoded to allow differentiation between individual DNA samples where the same variant was predicted. For *pinnable* variants, we used DNA that was predicted to contain the variant as the template. *Multiple* variants were verified in two ways; in the pool predicted to contain the variant and in every individual DNA sample predicted to contain the variant. *Singeltons* were verified by assaying every DNA sample in the row or column pool predicted to contain the variant.

Individually barcoded PCR samples were pooled and prepared for sequencing using standard Ion Torrent sample library preparation guidelines. The barcoded and pooled PCR libraries were run on an Ion Torrent Personal Genome Machine (PGM) and the resulting reads were evaluated against the reference genome.

## Supporting Information

File S1 Figure S1: Circos plot of filtered tracks, SNV calls and coverage over the region. The region of interest, a 250 kb region of chromosome 5 (5p15.33), is shown. Section A shows the genes TERT, CLPTM1L, BC034612, SLC6A3 and a portion of LPCAT1 in light green, with exons drawn in black. The grey and black tracks in section A highlight the repeat masked and self-chaining regions we excluded from analysis. Section B presents the positions of *pinnable* class SNVs in dark green, *multiple* SNVs in light green, *singletons* in grey and positions with some evidence but insufficient coverage (*missing coverage*) in black. Section C plots the depth of coverage over the region in dark green. The range of the plot is from 0× coverage at the outside of the light green band to 20,000× at the inside of the light green. Table S1: Predicted consequences of SNVs. Tables 2A, 2C, 2E and 2G show a breakdown for matrices 1, 2, 3 and 4 of the predicted consequences of *pinnable*, *multiple* and *singleton* SNVs. Tables 2B, 2D, 2F and 2H further list the transcription consequences for exonic variants from matrices 1 to 4 respectively. Table S2: Summary of indel class by matrix. The number of *Pinnable*, *Multiple* and *Singleton* indel variants identified is listed for each of the four 12×12 matrices. Also indicated is the percentage of each variant class that is catalogued in dbSNP. The number of total variants for each of the classes represents the total unique number of variants from all four matrices. Table S3: Predicted consequences of indels. The predicted consequences of the *pinnable*, *multiple* and *singleton* indel variants identified in matrices 1, 2, 3 and 4 are presented in Tables 3A, 3B, 3C and 3D.
(DOCX)
