# Supplementary material for: A Two-Dimensional Pooling Strategy for Rare Variant Detection on Next-Generation Sequencing Platforms
Source: PLoS One. 2014 Apr 11;9(4):e93455. doi: 10.1371/journal.pone.0093455 (PMC3984111; doi:10.1371/journal.pone.0093455)
Supplement: File S1 — Figure S1: Circos plot of filtered tracks, SNV calls and coverage over the region. The region of interest, a 250 kb region of chromosome 5 (5p15.33), is shown. Section A shows the genes TERT, CLPTM1L, BC034612, SLC6A3 and a portion of LPCAT1 in light green, with exons drawn in black. The grey and black tracks in section A highlight the repeat masked and self-chaining regions we excluded from analysis. Section B presents the positions of pinnable class SNVs in dark green, multiple SNVs in light green, singletons in grey and positions with some evidence but insufficient coverage (missing coverage) in black. Section C plots the depth of coverage over the region in dark green. The range of the plot is from 0× coverage at the outside of the light green band to 20,000× at the inside of the light green. Table S1: Predicted consequences of SNVs. Tables 2A, 2C, 2E and 2G show a breakdown for matrices 1, 2, 3 and 4 of the predicted consequences of pinnable, multiple and singleton SNVs. Tables 2B, 2D, 2F and 2H further list the transcription consequences for exonic variants from matrices 1 to 4 respectively. Table S2: Summary of indel class by matrix. The number of Pinnable, Multiple and Singleton indel variants identified is listed for each of the four 12×12 matrices. Also indicated is the percentage of each variant class that is catalogued in dbSNP. The number of total variants for each of the classes represents the total unique number of variants from all four matrices. Table S3: Predicted consequences of indels. The predicted consequences of the pinnable, multiple and singleton indel variants identified in matrices 1, 2, 3 and 4 are presented in Tables 3A, 3B, 3C and 3D. (DOCX) [file pone.0093455.s001.docx]

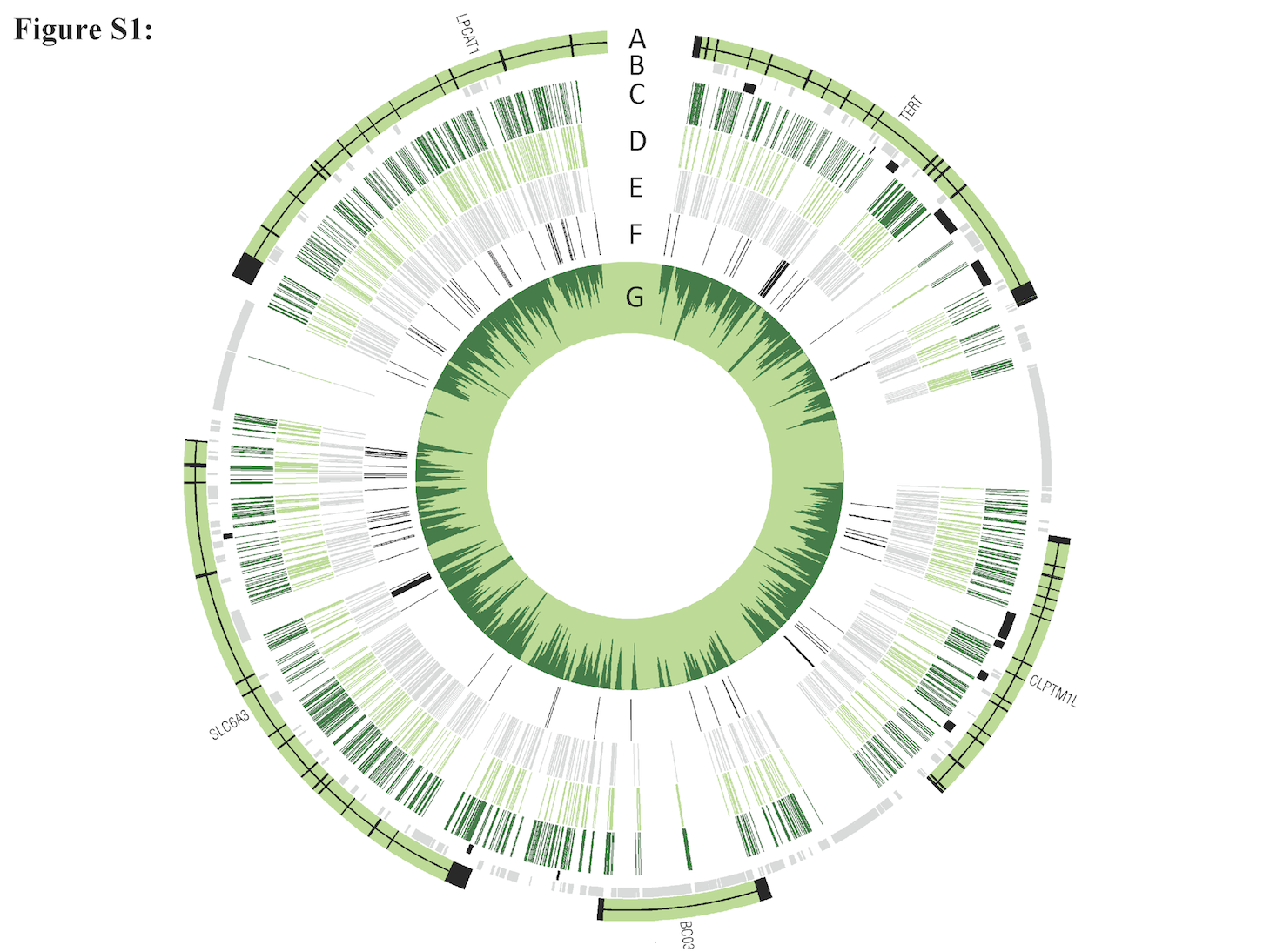


| **Table S1: Predicted consequences of SNVs** | | | |
| --- | --- | --- | --- |
| **Table S1A: Matrix 1 SNVs** | | |  |
|  |  |  |  |
| **Predicted Consequence** | **Pinnable** | **Multiple** | **Singleton** |
| downstream | 6 | 10 | 6 |
| exonic | 23 | 13 | 14 |
| exonic;splicing | 0 | 1 | 0 |
| intergenic | 63 | 116 | 67 |
| intronic | 266 | 425 | 238 |
| splicing | 0 | 0 | 1 |
| upstream | 3 | 3 | 8 |
| UTR3 | 10 | 23 | 7 |
|  |  |  |  |
|  |  |  |  |
| **Table S1B: Classification of Matrix 1 Exonic variants** | | | |
| **Predicted Consequence** | **Pinnable** | **Multiple** | **Singleton** |
| synonymous | 13 | 10 | 6 |
| nonsynonymous | 10 | 4 | 7 |
| stopgain | 0 | 0 | 1 |
|  |  |  |  |
|  |  |  |  |
|  |  |  |  |
| **Table S1C: Matrix 2 SNVs** | | |  |
|  |  |  |  |
| **Predicted Consequence** | **Pinnable** | **Multiple** | **Singleton** |
| downstream | 9 | 7 | 2 |
| exonic | 13 | 17 | 12 |
| exonic;splicing | 1 | 1 | 2 |
| intergenic | 91 | 121 | 30 |
| intronic | 297 | 437 | 120 |
| splicing | 0 | 0 | 0 |
| upstream | 2 | 10 | 1 |
| UTR3 | 12 | 20 | 12 |
|  |  |  |  |
|  |  |  |  |
| **Table S1D: Classification of Matrix 2 Exonic variants** | | | |
| **Predicted Consequence** | **Pinnable** | **Multiple** | **Singleton** |
| synonymous | 8 | 11 | 3 |
| nonsynonymous | 6 | 7 | 11 |
| stopgain | 0 | 0 | 0 |
|  |  |  |  |
|  |  |  |  |
| **Table S1E: Matrix 3 SNVs** | | |  |
|  |  |  |  |
| **Predicted Consequence** | **Pinnable** | **Multiple** | **Singleton** |
| downstream | 5 | 10 | 6 |
| exonic | 12 | 20 | 14 |
| exonic;splicing | 0 | 1 | 1 |
| intergenic | 80 | 128 | 40 |
| intronic | 298 | 487 | 175 |
| splicing | 0 | 0 | 0 |
| upstream | 0 | 13 | 3 |
| UTR3 | 13 | 21 | 6 |
|  |  |  |  |
|  |  |  |  |
| **Table S1F: Classification of Matrix 3 Exonic variants** | | | |
| **Predicted Consequence** | **Pinnable** | **Multiple** | **Singleton** |
| synonymous | 8 | 12 | 5 |
| nonsynonymous | 4 | 9 | 10 |
| stopgain | 0 | 0 | 0 |
|  |  |  |  |
| **Table S1G: Matrix 4 SNVs** | | |  |
|  |  |  |  |
| **Predicted Consequence** | **Pinnable** | **Multiple** | **Singleton** |
| downstream | 5 | 8 | 5 |
| exonic | 14 | 18 | 13 |
| exonic;splicing | 0 | 1 | 1 |
| intergenic | 58 | 123 | 43 |
| intronic | 222 | 438 | 160 |
| splicing | 0 | 0 | 0 |
| upstream | 2 | 12 | 4 |
| UTR3 | 11 | 21 | 5 |
|  |  |  |  |
|  |  |  |  |
| **Table S1H: Classification of Matrix 4 Exonic variants** | | | |
| **Predicted Consequence** | **Pinnable** | **Multiple** | **Singleton** |
| synonymous | 10 | 10 | 5 |
| nonsynonymous | 4 | 9 | 9 |
| stopgain | 0 | 0 | 0 |

| **Table S2: Summary of indel class by matrix** | | |  |
| --- | --- | --- | --- |
|  |  |  |  |
| **Matrix** | **Pinnable Variants (% dbSNP)** | **Multiple Variants (% dbSNP)** | **Singleton Variants (% dbSNP)** |
| 1 | 24 (4.17%) | 58 (18.97%) | 52 (0.00%) |
| 2 | 23 (8.70%) | 67 (16.42%) | 38 (2.63%) |
| 3 | 18 (5.56%) | 72 (19.44%) | 38 (0.00%) |
| 4 | 17 (0.00%) | 67 (19.40%) | 36 (0.00%) |
| Total unique | 70 (4.29%) | 78 (16.6%) | 150 (0.67%) |

| **Table S3: Predicted consequences of indels** | | | |
| --- | --- | --- | --- |
| **Supplemental Table S3A: Matrix 1 Indels** | |  |  |
|  |  |  |  |
| **Predicted Consequence** | **Pinnable** | **Multiple** | **Singleton** |
| downstream | 0 | 1 | 0 |
| frameshift substitution | 0 | 0 | 0 |
| intergenic | 6 | 12 | 9 |
| intronic | 18 | 45 | 40 |
| upstream | 0 | 0 | 0 |
| UTR3 | 0 | 0 | 3 |
|  |  |  |  |
|  |  |  |  |
|  |  |  |  |
| **Table S3B: Matrix 2 Indels** | |  |  |
|  |  |  |  |
| **Predicted Consequence** | **Pinnable** | **Multiple** | **Singleton** |
| downstream | 0 | 1 | 2 |
| frameshift substitution | 0 | 0 | 1 |
| intergenic | 2 | 11 | 8 |
| intronic | 20 | 52 | 27 |
| upstream | 0 | 0 | 0 |
| UTR3 | 1 | 3 | 0 |
|  |  |  |  |
|  |  |  |  |
|  |  |  |  |
| **Table S3C: Matrix 3 Indels** | |  |  |
|  |  |  |  |
| **Predicted Consequence** | **Pinnable** | **Multiple** | **Singleton** |
| downstream | 0 | 2 | 0 |
| frameshift substitution | 0 | 0 | 2 |
| intergenic | 1 | 10 | 7 |
| intronic | 16 | 60 | 28 |
| upstream | 0 | 0 | 1 |
| UTR3 | 1 | 0 | 0 |
|  |  |  |  |
|  |  |  |  |
|  |  |  |  |
| **Table S3D: Matrix 4 Indels** | | |  |
|  |  |  |  |
| **Predicted Consequence** | **Pinnable** | **Multiple** | **Singleton** |
| downstream | 0 | 1 | 1 |
| frameshift substitution | 0 | 0 | 2 |
| intergenic | 2 | 11 | 4 |
| intronic | 15 | 52 | 27 |
| upstream | 0 | 0 | 0 |
| UTR3 | 0 | 3 | 2 |
|  |  |  |  |

**Text S1: Variant Classification Pseudocode**

*# first, align and filter the reads from the row and column pools to create bam and pileup files*

For each fastq file (or pair of fastq files, for paired end sequencing)

Map reads to the human genome using aligner of choice

Realign reads using GATK

Remove reads with low MAPQ (< 30 for Novoalign, < 20 for BWA)

Remove reads with > 2 mismatches

Generate pileup format from the bam files (samtools mpileup)

*# next, collect the total depth, number of reads supporting each variant, and start point bias for* every position in the targeted region in order to classify the variants

For each pileup file (one per row or column pool)

For each genomic position in the targeted region and not in UCSC’s repeat mask or segmental duplication tracks

Determine the total depth of coverage (bases with quality >= 30)

For each non-reference base or indel supported by a read at the position

Determine the depth of coverage supporting the variant (q >= 30)

Calculate Start Point Bias Metric score (total variant depth / max variant depth from one start point – see Methods)

Classify the variant using the Single Pool Classification Subroutine (next)

*# Single Pool Classification Subroutine*

If total depth is > 200 bp and Start Point Bias Metric score is > 1.25

If < 0.1% of reads support the variant

Classify variant as “Confident Reference”

Else if < 0.5% of reads support the variant

Classify variant as “Potential Reference”

Else if >= 1% of reads support the variant

Classify variant as “Confident Variant”

Else if >= 0.5% of bases support a variant

Classify variant as “Potential Variant”

Else (sequence coverage is insufficient)

Classify position as “Insufficient Coverage”

*# finally, consider all of the row and column pools to classify each variant’s status in the matrix*

For each variant with at least one “Confident Variant” call

If the number of pools with “Insufficient Coverage” is < 3

If there is one or more rows with a “Confident Variant” and/or “Potential Variant” and exactly one column with a “Confident Variant” or “Potential Variant”

Classify variant as “Pinnable”

Else if there is one or more columns with a “Confident Variant” and/or “Potential Variant” and exactly one row with a “Confident Variant” or “Potential Variant”

Classify variant as “Pinnable”

Else if there is more than one row and more than one column with a “Confident Variant” and/or “Potential Variant”

Classify variant as “Multiple”

Else if there are zero “Confident Variant” or “Potential Variant” calls in either the rows or the columns

Classify variant as “Singelton”

Else (3 or more pools with insufficient coverage)

Classify variant as “Missing Coverage”
